# Compartmentalization of DHCR7 links 7-DHC metabolism to B-ring unsaturated steroid synthesis in the equine fetal gonad

**DOI:** 10.1210/endocr/bqag083

**Published:** 2026-07-25

**Authors:** Katarzyna Malin, Alan J Conley, Margo Verstraete, Jamie Norris, Ya-Chen Hsu, William Holl, Machteld van Heule, Flávia Vieira, Kirsten E Scoggin, Barry Ball, Kazuki Takahashi, Alejandro de la Fuente, John W Newman, Mariano Carossino, Pouya Dini

**Affiliations:** Department of Population Health and Reproduction, School of Veterinary Medicine, University of California, Davis, CA 95616, USA; Department of Population Health and Reproduction, School of Veterinary Medicine, University of California, Davis, CA 95616, USA; Department of Population Health and Reproduction, School of Veterinary Medicine, University of California, Davis, CA 95616, USA; Department of Morphology, Imaging, Orthopedics, Rehabilitation and Nutrition, Faculty of Veterinary Medicine, Ghent University, 9820 Merelbeke, Belgium; Department of Population Health and Reproduction, School of Veterinary Medicine, University of California, Davis, CA 95616, USA; West Coast Metabolomics Center, Genome Center, University of California, Davis, CA 95616, USA; Department of Pathobiological Sciences and Louisiana Animal Disease Diagnostic Laboratory, School of Veterinary Medicine, Louisiana State University, Baton Rouge, LA 70803, USA; Department of Population Health and Reproduction, School of Veterinary Medicine, University of California, Davis, CA 95616, USA; Department of Morphology, Imaging, Orthopedics, Rehabilitation and Nutrition, Faculty of Veterinary Medicine, Ghent University, 9820 Merelbeke, Belgium; Department of Population Health and Reproduction, School of Veterinary Medicine, University of California, Davis, CA 95616, USA; Department of Veterinary Science, Gluck Equine Research Center, University of Kentucky, Lexington, KY 40546, USA; Department of Veterinary Science, Gluck Equine Research Center, University of Kentucky, Lexington, KY 40546, USA; Department of Population Health and Reproduction, School of Veterinary Medicine, University of California, Davis, CA 95616, USA; Department of Population Health and Reproduction, School of Veterinary Medicine, University of California, Davis, CA 95616, USA; West Coast Metabolomics Center, Genome Center, University of California, Davis, CA 95616, USA; Department of Nutrition, University of California Davis, Davis, CA 95616, USA; Obesity and Metabolism Research Unit, USDA Western Human Research Center, Davis, CA 95616, USA; Department of Pathobiological Sciences and Louisiana Animal Disease Diagnostic Laboratory, School of Veterinary Medicine, Louisiana State University, Baton Rouge, LA 70803, USA; Department of Population Health and Reproduction, School of Veterinary Medicine, University of California, Davis, CA 95616, USA

**Keywords:** equilin, 7-dehydrocholesterol, DHCR7, smith-Lemli-Opitz, fetal gonad, DHCEO

## Abstract

The endocrinology of equine pregnancy is unique, but many of its unusual phenomena closely resemble pregnancy in women. Placental estrone synthesis uses fetal adrenal androgen precursors in women, but from the fetal gonads in mares, peaking midway through the 11-month gestation. Unique to equine pregnancies, other estrogens like equilin, typified by an unsaturated B-ring sterol structure, are synthesized from 7-dehydrocholesterol (7-DHC), the immediate precursor to cholesterol, without cholesterol formation, peaking later in gestation. Currently, the spatial and temporal regulation underlying the redirection of 7-DHC from cholesterol to B-ring unsaturated steroid synthesis in equine fetal gonads remains unknown. Here, we investigate the developmental dynamics of the equine fetal gonads from the fourth to eleventh gestational month using RNA sequencing, immunofluorescence imaging, RNAScope, and gas chromatography–mass spectrometry. Our results suggest that placental estrone synthesis correlates with the expression of the last enzyme in the cholesterol synthesis pathway, 7-dehydrocholesterol reductase (*DHCR7*) in the fetal gonad, and its downregulation may drive the accumulation of 7-DHC and the synthesis of B-ring unsaturated steroids. B-ring unsaturated steroid secretion is the hallmark of Smith–Lemli–Opitz Syndrome in humans, resulting from mutations in the gene encoding *DHCR7* in affected patients. This naturally occurring phenomenon in equine pregnancies may offer a unique comparative model for understanding the metabolic consequences of 7-DHC accumulation in these patients and a platform for drug development.

In both human and equine pregnancies, a distinct fetoplacental interplay is utilized for placental estrogen synthesis. The equine and primate placentae, although competent to aromatize androgens to estrogens, have a similar, limited capacity for producing androgens (1). In primates, the fetal adrenal “fetal zone” produces large quantities of dehydroepiandrosterone for eventual placental aromatization (2, 3). Analogously, the interstitial compartment of the equine fetal gonad has evolved to synthesize androgen substrates for the placental aromatization (3, 4), and estrogen synthesis increases as both tissues experience rapid growth. In the fetal gonad of the horse, this occurs at the end of the second trimester, when it is 10-fold larger than it is at birth (5). Maternal estrogen concentrations increase and decline during pregnancy in concert with the growth and regression of the equine fetal gonads (6-8).

In mares, estrone (a classical, cholesterol-derived steroid) concentration peaks at approximately gestational day 140, whereas equilin ([9*S*,13*S*,14*S*]-3-hydroxy-13-methyl-9,11,12,14,15,16-hexahydro-6*H*-cyclopenta[a]phenanthren-17-one) (a B-ring unsaturated steroid derived from a direct cholesterol precursor, 7-dehydrocholesterol [7-DHC]), reaches maximal concentration around days 210 to 240 and declines toward term (4, 6, 9-12) (molecular formulas used in the manuscript (13) are showed in Fig. S1 (14)). Earlier studies showed the steroidogenic capability of the equine fetal gonads, and identified the precursors to both estrone and equilin in the tissues (15-17), further confirming that the gonads are the site of synthesis for both steroid families. It is not known how the equine fetal gonad regulates the conversion of both 7-DHC and cholesterol to steroids at the gene or protein expression level, spatially or temporally.

Based on the previously reported shifts in the maternal estrone and equilin levels, we hypothesized that 7-dehydrocholesterol reductase (*DHCR7*), the enzyme catalyzing the final step in the Kandutsch–Russell cholesterol synthesis pathway, is selectively downregulated within the interstitium of the equine fetal gonad. We infer that a relative decrease in this terminal enzyme would promote the accumulation of 7-DHC and fuel increased B-ring unsaturated steroid synthesis. The development of the equine fetal gonad and its interstitial (steroidogenic) compartment was investigated in tissues collected between the 4th and 11th gestational month (GM) by RNA sequencing (RNAseq), in situ hybridization (ISH), immunohistochemistry (IHC), immunofluorescence (IF), and gas chromatography–mass spectrometry (GC-MS). Our findings confirmed the hypothesis, as *DHCR7* was expressed differentially both spatially and temporally in the fetal gonads, with the highest but spatially restricted expression in the 4th GM and 7-DHC accumulated in the equine fetal gonad but not control samples from the equine fetal adrenal glands. We concluded that the selective downregulation of *DHCR7* likely determines the availability of classical and B-ring unsaturated precursors, leading to the development of an interstitial compartment that is capable of metabolizing high 7-DHC levels. Synthesis of equilin has been documented in the Smith–Lemli–Opitz syndrome (SLOS), where mutations in *DHCR7* lead to DHCR7 deficiency and 7-DHC accumulation, leading to severe developmental defects (18-20). Thus, our results reveal a potential framework for B-ring unsaturated steroid formation, a natural model with possible implications for SLOS for research, drug development, and in vivo testing.

## Results

**Figure 1:**
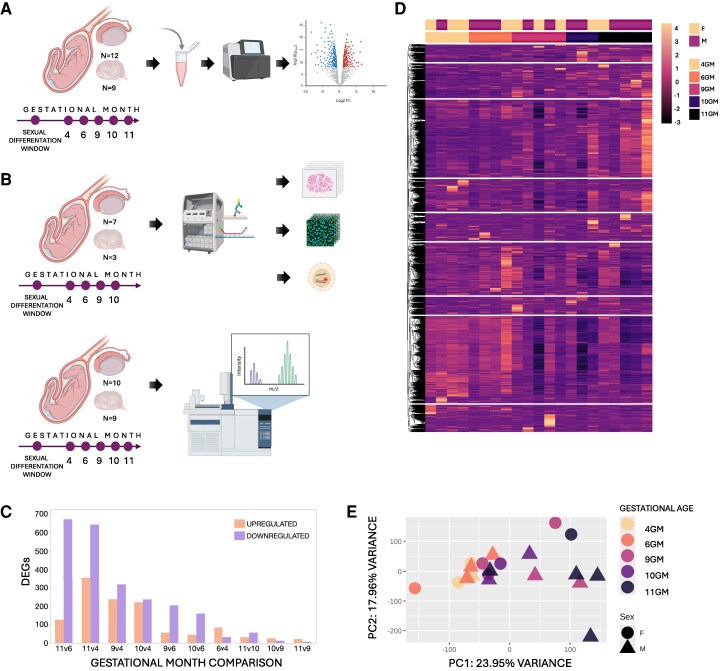
Study design and the global transcriptomic analysis of the equine fetal gonad. (A) Samples from the 4th to 11th GM were used for RNA sequencing and (B) tissue histology and IHC, and GC-MS. *Created with Biorender*. (C) Differentially expressed genes (DEGs) in each comparison reveal the highest differential expression between the oldest and the youngest samples. (D) Clustering of DEGs based on fetal age and sex (*k*-means). (E) Principal component analysis (PCA) reveals clustering based on age, especially in the earlier stages of pregnancy.

First, we aimed to identify and characterize the interstitial cell populations of the equine fetal ovary and testis. Although histological and immunohistochemical studies have been performed before (21-25), neither presented a clear model of spatial and temporal changes, linking morphology to function. We used a total of 21 gonads from the 4th to the 11th GM for histological evaluation, RNAseq, RNAscope, IF, IHC, and GC-MS (Fig. 1A and 1B).

### Global transcriptomic dynamics of the fetal gonad

**Figure 2:**
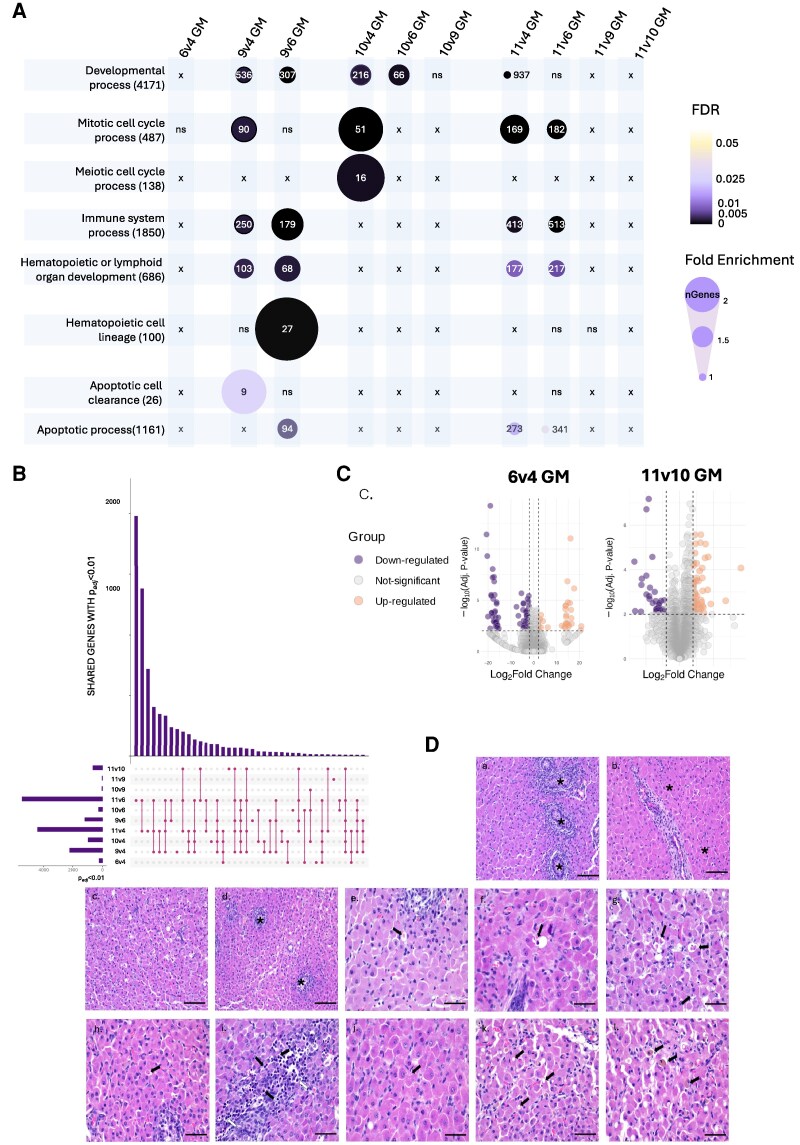
Transcriptomic and histological analysis of the equine fetal gonad. (A) Gene ontology analysis showed involvement of the immune system processes, hematopoietic development, and apoptosis in the fetal gonad development. (B) Enrichment analysis. (C) A low number of differentially expressed genes was found comparing 4th and 6th GM and between 10th and 11th GM; therefore, no significant GO was identified. (D) (a-d) Histologic features of the developing fetal gonad. The fetal gonad is composed primarily by interstitial cells characterized by abundant eosinophilic cytoplasm and round nuclei with open chromatin. In the developing testis (a and d, 4GM and 10GM), there are primitive seminiferous tubules with a lack of histologically distinct sperm lineage cells (asterisk). The fetal ovary (b and c, 4GM and 9GM) is composed of interstitial cells and vasculature (asterisk). H&E. Bar 100 µm. (e-h) Apoptosis in the developing gonad during gestation. (e and f) Fetal testis from a 4GM and 6GM fetus, respectively. Infrequent interstitial cells are shrunken, individualized, and rounded with cytoplasmic hypereosinophilia and nuclear pyknosis (apoptosis) (arrow). (g) Fetal ovary from a 9-month gestational age fetus. There are frequent clusters of apoptotic interstitial cells (arrows). (h) Fetal testis from a 10 GM fetus. Apoptotic interstitial cells are sporadic (arrow). H&E. Bar 50 µm. (i-l) Additional histologic features of the developing gonad. (i) The parenchyma has sporadic foci of extramedullary hematopoietic precursor cells (arrows, 6GM, fetal testis). (j-k) Many interstitial cells contain small (j) to large (k), well-delimited, round, cytoplasmic lipid vacuoles (arrows; fetal testis from a 6GM and 10GM fetus). (l) Multiple interstitial macrophages contain cytoplasmic orange-brown, granular to globular ceroid pigment (arrows; fetal testis from 10GM fetus). H&E. Bar 50 µm.

The highest degree of differential gene expression was seen between the 11th and 4th GM, with *n* = 1001 differentially expressed genes (DEGs; Log_2_FC ≥ |2|, *P*_adj_ = .01; File S1 (26). The least differential expression was seen in the comparisons between the 9th, 10th, and 11th GMs (Fig. 1C-1E). In the 6th vs 4th GM, genes implicated in hematopoiesis (*BCL11A, VENTX*) (27, 28), immune response (*CLEC4G*) (29) and hormonal response (*OXTR, TRHDE, MC3R*) (30-32) were downregulated. In contrast, genes associated with meiosis and germline development, such as *MEIKIN*, *SPAG6*, and *ZAR1* (33-35), were upregulated. In the third trimester, in the 11th vs 9th GM, immune system and erythroid-cell-associated genes (*HBB, HBA2, HBA, SLC4A1, GYPA*) (36-38) were expressed differentially; MiR-708 and *RAX*, associated with apoptosis (39, 40), were upregulated. Gene ontology analysis (File S2 (41)) of the DEGs in the oldest (11th GM) vs the youngest (4th GM) fetal gonads showed enrichment in mitotic cell processes (Fig. 2A; DEGs shown in Fig. 2B and 2C). The significantly upregulated genes were related to immune system (*CD3E, CD3G, SKAP1, ZAP70, CD3D, TRAT1*) (42-45), and hematopoiesis (*CSF2RB, GFI1, ZAP70, CXCL12*) (46-49), with downregulation in genes linked to cell cycle and meiosis (*FOXM1*, cyclin B family (*CCNB1, CCNB2, CCNB3*) (50, 51), mitotic checkpoint kinases (*BUB1, BUB1B, TTK*) (52), and mitotic kinesins and kinases (*NEK2, KIF11, KIF20A, KIF14, KIF18A, KIF4A*) (53, 54).

### Histology of interstitial hypertrophy, hematopoiesis, and apoptosis

To further investigate the gonadal hypertrophy and hyperplasia, potential immune system involvement, apoptosis, and hematopoiesis-related gene expression, samples from the 4th to 10th GM were evaluated histologically using hematoxylin and eosin (H&E) staining (Fig. 2D). The hypertrophy and hyperplasia of the equine fetal gonad were previously attributed to the interstitial compartment development (5), and the presence of interstitial cells in the gonads was reported as early as day 60, approximately a month before estrogens first begin to rise (55, 56). The fetal gonadal interstitium was composed of round to polygonal cells with distinct cell borders having abundant eosinophilic, variably vacuolated cytoplasm and round nuclei with open chromatin, separated by small capillaries (Fig. 2Da-d). Rare interstitial cells had hypereosinophilic cytoplasm and pyknotic to karyorrhectic nuclei across GM (apoptosis; Fig. 2De-h). Scattered extramedullary hematopoietic precursor cells were seen with more prominent clustering in the interstitium around larger blood vessels or around the developing seminiferous tubules in fetal testes (Fig. 2Di). These extramedullary hematopoietic cells either had bright eosinophilic cytoplasm with condensed nuclei (consistent with erythroid lineage) or amphophilic to basophilic cytoplasm with larger, less condensed nuclei (consistent with myeloid lineage). Intracytoplasmic vacuoles were found across all ages but were larger in the older samples and were variably tinted with golden yellow pigment (ceroid) (Fig. 2Dj-k). With increasing GM, there were more prevalent macrophages with expanded cytoplasm containing similar golden yellow pigment (2Dl).

### Developmental regulation of cholesterol-pathway genes

**Figure 3:**
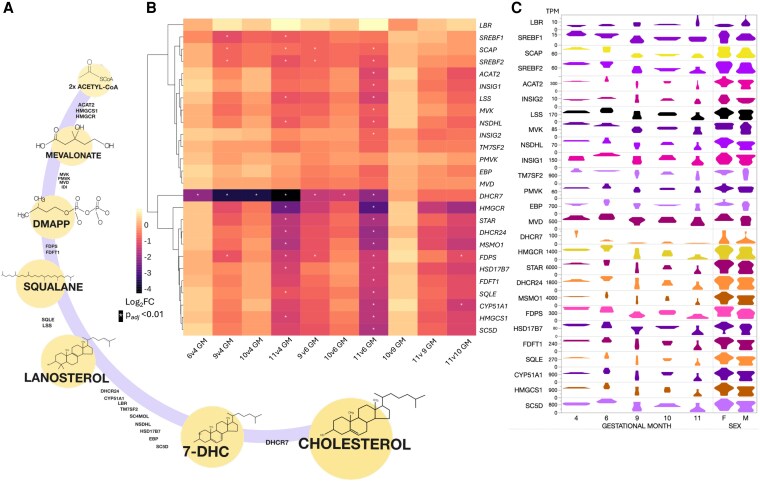
Expression of genes encoding the enzymes of cholesterol biosynthesis pathway. (A) Cholesterol biosynthesis pathway (Kandutsch–Russel pathway). (B) Changes in expression in the cholesterol biosynthesis pathway throughout the pregnancy suggest that DHCR7 acts as the rate-limiting enzyme. (C) Quantified expression shows that although most enzymes upstream of *DHCR7* had the numerically highest expression in the 6th month, they were not differentially expressed.

To elucidate whether the decline in estrone seen at the end of the second trimester in the mare (6) was correlated solely with *DHCR7* expression pattern, and to exclude any other metabolic changes that could lead to this outcome, we evaluated the expression of genes encoding enzymes identified to participate in cholesterol synthesis from acetyl-CoA (Fig. 3A; Table S1 (57)). Such a mechanism would lead to the accumulation of 7-DHC for equilin synthesis. As estrone and equilin typically reach similar peak concentrations (6), even if at different times, we also expected a decline in overall expression in the pathway in some cells, reflecting the decrease in estrogen synthesis toward the end of gestation. The expression of major regulatory protein encoding genes, *SCAP, SREBF2*, *INSIG1* and *INSIG2* (58, 59), and *STAR*, which facilitate transport of cholesterol into the mitochondria (60), were also evaluated (Fig. 3B and 3C; Table S2 (61)); however, no significant differential expression was seen between the time points except for DHCR7 which was the highest in the 4th and 6th month, in agreement with our hypothesis (Log_2_FC = −4.28 in the 11th vs 4th GM, *P*_adj_ = 4.07 × 10^−30^), and *HMGCR* was differentially expressed in the 11th GM compared to the 4th GM (Log_2_FC = −2.51, *P*_adj_ = .003) and 6th GM (Log_2_FC = −2.84, *P*_adj_ = .0002), suggesting a pivotal role in the decline in estrogen availability peri-parturition, alongside the regression of the size of the fetal gonad.

### Steroidogenic enzyme localization

**Figure 4:**
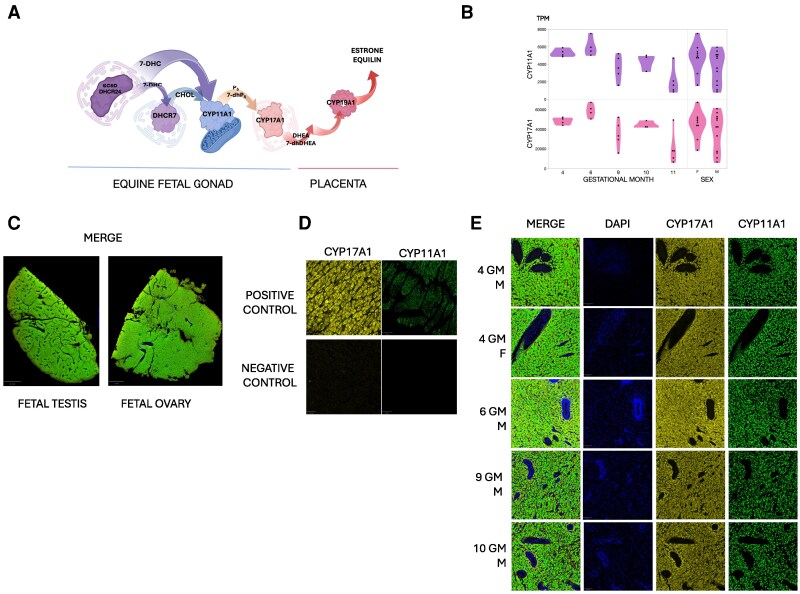
The steroidogenic activity of the equine fetal gonad. (A) Synthesis pathway of estrone and equilin. *Created with Biorender.* (B) RNA sequencing shows no differential expression in CYP11A1 and CYP17A1 throughout the pregnancy and with regard to fetal sex (M = male; F = female). (C) The equine fetal gonad acts as a steroidogenic unit with uniform CYP11A1 and CYP17A1 expression (scale bar = 2 mm (fetal testis); scale bar = 1 mm [fetal ovary]). (D) Positive (adult adrenal cortex) and negative (adult adrenal medulla) controls for CYP11A1 and CYP17A1 antibodies (scale bar = 50 μm). (E) Spatial expression of CYP11A1 and CYP17A1 (IF), DAPI staining nuclei from the 4th to 10th gestational month (scale bar = 100 μm).

Previously, ultrastructural studies of the equine fetal gonad interstitium confirmed the presence of abundant smooth endoplasmic reticulum, mitochondria with cristae, and the presence of lipid material with a well-developed Golgi apparatus, which are considered characteristic of steroidogenic cells (62). However, 3β-hydroxysteroid dehydrogenase, a broadly used marker of steroidogenic cells, was not identified within this compartment in the same report, in agreement with trace amounts of progesterone found in the gonad in later studies (4, 62). We thus sought to identify regions potentially capable of steroid synthesis, defined by CYP11A1 expression (C21, pregnane synthesis), and CYP17A1 expression (C19, androstane synthesis) (immediate upstream and downstream steps in the cholesterol biosynthetic pathway and steroidogenesis centered on DHCR7 seen in Fig. 4A). The temporal expression of these genes was evaluated in RNAseq, and although *CYP17A1* was among the most highly expressed genes in all age groups, no differential expression was identified (Fig. 4B; Table S3 (63)). In IF, in all male and female samples, the presence of both proteins overlapped and matched the steroidogenic cell morphology (Fig. 4C); however, seminiferous tubules in the testis and the vascular components of the ovary were directly circumscribed by cells negative for both proteins (Fig. 4D and 4E). Thus, the interstitial cells are likely equally capable of synthesizing estrogen precursors for the placenta; furthermore, CYP11A1 and CYP17A1 are likely not limiting their synthesis, pointing to the changes in size of the gonad as the rate-limiting factor.

### Spatial mapping of DHCR7 downregulation

**Figure 5:**
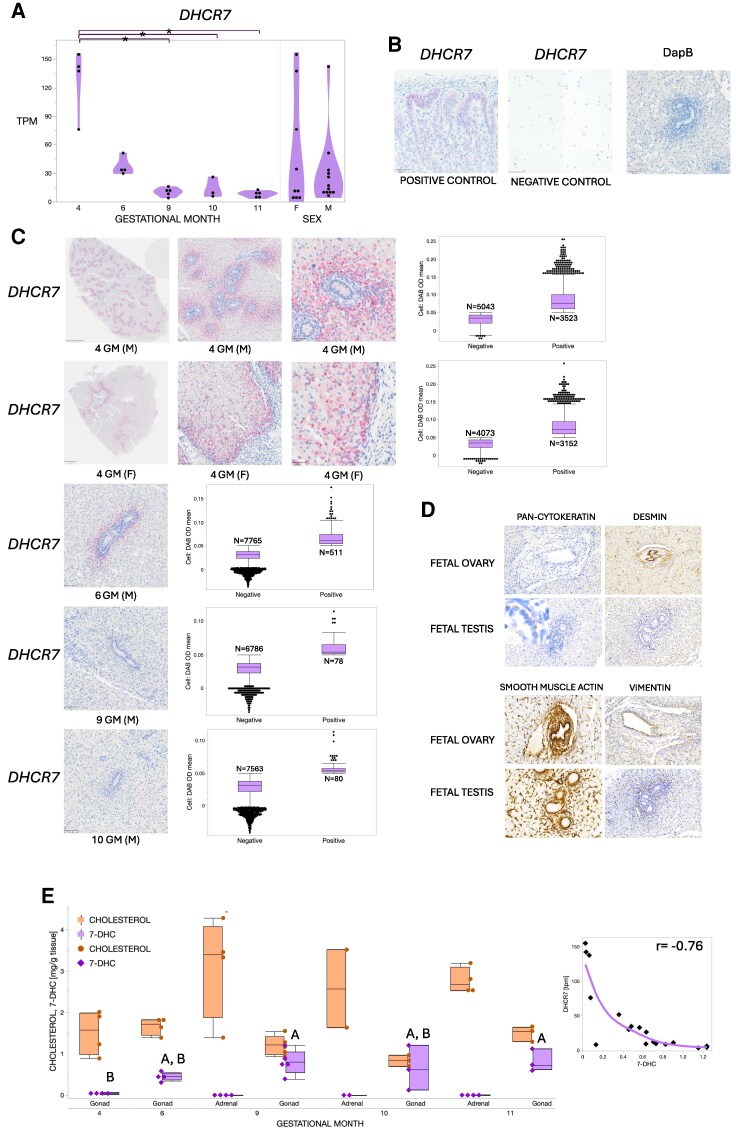
Fetal ovary and fetal testis express DHCR7 differentially spatially and temporally. A. *DHCR7* expression (RNAseq,) was the highest in the 4th GM in correlation with mare plasma estrone shown previously. B. Positive control for *DHCR7 (red)* RNAscope™ ISH (adult equine adrenal gland cortex); negative control for *DHCR7* RNAscope™ ISH (adult equine adipose tissue); DapB. (Scale bar = 100 μm). C. Spatial expression of *DHCR7* (RNAscope™ ISH) reveals spatial compartmentalization focused around the ovarian vasculature and seminiferous tubules (Scale bars (4GM, M, left to right = 2 mm; 100 μm; 50 μm), (4GM, F, left to right = 1 mm; 100 μm; 50 μm), 6GM = 100 μm, 9GM = 100 μm, 10GM = 100 μm). D. Identification of the non-steroidogenic cell populations within the equine fetal gonad (immunohistochemistry). Pan-cytokeratin expression is restricted to the cytoplasm of primitive Sertoli cells in the seminiferous tubules (inset). Stromal cells surrounding ovarian vasculature and testicular developing seminiferous tubules (4GM) are characterized by expression of desmin, vimentin and smooth muscle actin, suggesting these represent smooth muscle cells or myofibroblasts. (E) Concentrations of cholesterol and 7-dehydrocholesterol (7-DHC) in equine fetal gonads and adrenals across gestation. Sterol abundance was quantified in fetal gonads collected at the 4th, 6th, 9th, 10th, and 11th gestational months and in fetal adrenals collected at the 9th, 10th, and 11th gestational months, which served as steroidogenic control tissues. Pearson correlation between *DHCR7* expression (TPM) and 7-dehydrocholesterol concentration (mg/g tissue) across samples. Pearson's correlation coefficient: *r* = −0.76, *P* = .0002.

Lastly, we reasoned that the simultaneous decline in *DHCR7* expression (Fig. 5A) and the reported estrone concentration, together with the increase in the volume of the interstitial compartment and equilin's concentration in the second half of the pregnancy (6, 64), suggested that the two hormones may be synthesized in separate spatial compartments. Thus, *DHCR7* gene expression was investigated by RNAscope™ ISH (Fig. 5B and 5C). At the 4th GM, the interstitial cells expressed *DHCR7* most strongly around the supporting interstitium circumscribing the vasculature in the fetal ovary and seminiferous tubules in the fetal testis with weaker and scattered expression in other areas, creating two spatial compartments with strong and weak *DHCR7* expression (Fig. 5C). We further phenotyped these circumscribing cells and determined these likely to represent smooth muscle cells or myofibroblasts based on expression of α-Smooth Muscle Actin, Vimentin, and Desmin, with lack of cytokeratin expression, indicating mesenchymal origin (Fig. 5D). In the 6th month, the population of DHCR7-strong-positive cells declined in agreement with RNAseq findings. All interstitial cells were weakly positive in all 9th- and 10th-month samples, without spatial differential expression, and no significant differences between sexes. Thus, in agreement with our initial hypothesis, the equine fetal gonad downregulates *DHCR7* spatially and temporally, likely leading to the accumulation of 7-DHC, allowing for compartmentalized synthesis of equilin precursors.

### GC–MS quantification of cholesterol and 7-dehydrocholesterol

Cholesterol and 7-DHC concentrations were quantified by GC–MS in fetal gonads collected at 4, 6, 9, 10, and 11 months of gestation and in fetal adrenals collected at 9, 10, and 11 months, which served as steroidogenic tissue controls (Fig. 5E). In fetal gonads, mean cholesterol concentrations measured at 4 months were 1.1550 mg/g tissue, 1.708 mg/g at 6 months, 1.232 mg/g at 9 months, 0.857 mg/g at 10 months, and 1.533 mg/g at 11 months. Mean 7-DHC concentrations in fetal gonads measured 0.048 mg/g at 4 months, 0.466 mg/g at 6 months, 0.815 mg/g at 9 months, 0.667 mg/g at 10 months, and 0.838 mg/g at 11 months. The 7-DHC:cholesterol ratio in gonadal tissue increased across gestation (*P* ≤ .02), with the highest ratio observed in a 10-month female fetus (1.436:1) and the lowest in a 4-month female fetus (0.012:1). In contrast to gonadal tissue, the adrenal cholesterol:7-DHC ratio remained at or less than 1:0.03 across samples and gestation stages. Furthermore, *DHCR7* expression demonstrated a significant inverse correlation with 7-DHC accumulation (*r* = −0.76, *P* = .0002), indicating that reduced *DHCR7* expression was associated with increased accumulation of its substrate in vivo.

## Discussion

**Figure 6:**
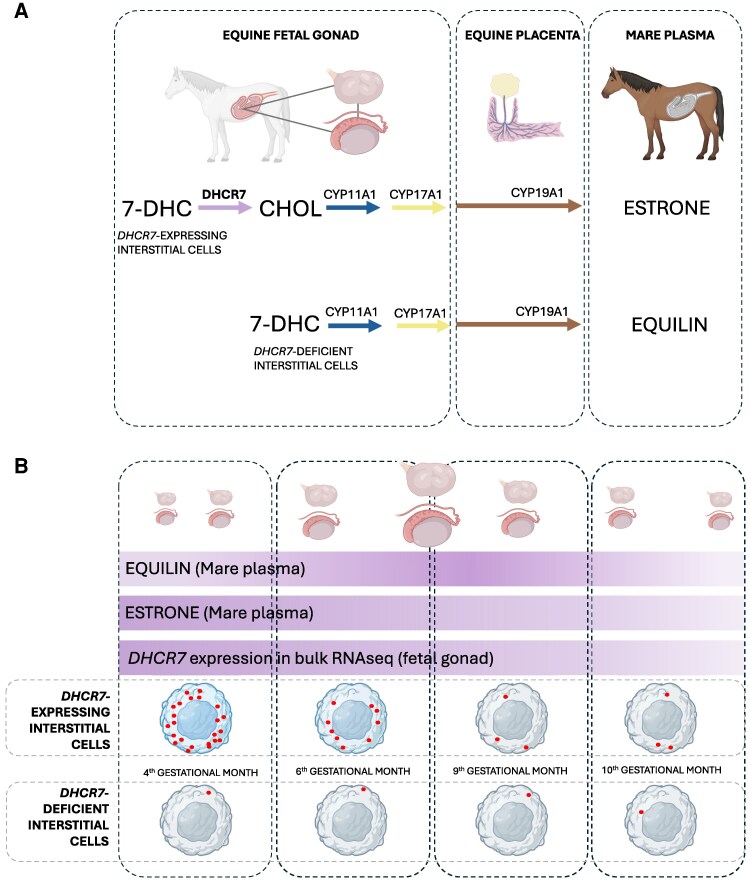
Proposed model of the steroidogenic activity of the equine fetoplacental unit from 4th to 11th gestational month. (A) The equine fetal gonad synthesizes 7-DHC in all steroidogenic cells, but not all steroidogenic cells express DHCR7, the terminal enzyme of the Kandutsch–Russel cholesterol synthesis pathway. The *DHCR7*-deficient subpopulation synthesizes precursors to equilin, namely 7-dehydropregnenolone and 7-dehydro-dehydroepiandrosterone. The steroidogenic population expressing *DHCR7* synthesizes pregnenolone and dehydroepiandrosterone from cholesterol. These steroids are then converted to equilin and estrone, respectively, and are detectable in the mare's plasma and urine. *Created with Biorender.* (B) The developmental trajectory of the equine fetal gonad steroidogenic cells with regards to *DHCR7* expression. The gonad reaches its maximal size in correlation with equilin's peak in the mare plasma circa 8th gestational month. *DHCR7* expression in the gonadal steoirogenic cells (red dots) is correlated with mare plasma estrone. Toward the end of the pregnancy, the steroidogenic cells of the gonad display uniformly low *DHCR7* expression. *Created with Biorender*.

Here, we report that during normal equine pregnancy, the equine fetal gonads downregulate *DHCR7* spatially and temporally in the steroidogenic interstitial compartment, likely orchestrating the shift from classical estrone to 7-DHC-derived B-ring unsaturated equilin in the mare plasma (simplified models shown in Fig. 6A and 6B). As a result, 7-DHC accumulates in the fetal gonads as they develop in the second half of pregnancy, and decreases as apoptosis induces regression of the interstitial tissue in late gestation. *DHCR7* was expressed differentially across pregnancy as demonstrated by RNAseq, and spatially as shown by RNAscope, with zonal expression being highest only near the smooth muscle cells/myofibroblast populations around the seminiferous tubules in the fetal testis and the vascular component of the ovary in the second trimester. Our findings suggest that early in gestation, higher *DHCR7* expression in the interstitial cells channels 7-DHC toward cholesterol and estrone synthesis, but as the interstitial tissue develops it is relatively deficient in *DHCR7*. As a result, 7-DHC accumulates and is diverted into B-ring unsaturated steroid synthesis, favoring equilin synthesis. The zonation became less prominent with advancing fetal age, and by the 9th GM, the expression of *DHCR7* across the interstitium was uniformly low and 7-DHC reached peak concentrations.

The steroidogenic fate of 7-DHC in the equine fetal gonad requires its transportation from the endoplasmic reticulum (65), without conversion to cholesterol, to the inner mitochondrial membrane, where it can become a substrate for CYP11A1 (65). Increase in 7-DHC accumulation was in agreement with previously found equilin levels in the mare plasma (10). Of note, 7-DHC is prone to oxidation to DHCEO ([(3*S*,5*R*,10*R*,13*R*,17*R*)-5-hydroxy-10,13-dimethyl-17-(6-methylheptan-2-yl)-6-oxo-2,3,4,7,8,9,11,12,14,15,16,17-dodecahydro-1*H*-cyclopenta[a]phenanthren-3-yl] acetate.), a cytotoxic oxysterol (66-68). Consequentially, DHCEO was proposed as the critical mediator of pathophysiology in embryonic Dhcr7-KO mice, and shown to reduce the viability of Neuro2a cells in culture (69, 70). Deficiency of DHCR7 also induced cell apoptosis independently of cholesterol in squamous cell carcinoma (71). Furthermore, loss of DHCR7 in vitro was shown to lead to accumulation of DHCEO in human cortical neural precursors (67). Thus, in SLOS patients, a lack of DHCR7 results in cholesterol deficiency (72), 7-DHC accumulation (73), and potentially, DHCEO-induced cytotoxicity. By analogy, we hypothesize that oxysterol formation may also be responsible for the regression of the interstitial compartment of the equine fetal gonad, directly mimicking SLOS. Future studies investigating DHCEO tissue concentrations will be necessary to properly explore this hypothesis.

Loss of function in *DHCR7* is particularly challenging to study, as earlier attempts to treat rodents with a DHCR7 inhibitor or to induce a loss-of-function mutation resulted in neonatal lethality, limiting research to the embryonic period (68, 74-78). Short-term inhibitor treatment of the Neuro2a cell line has valuable, albeit limited, application (79). Recently, the development of the iPCS lines from patient fibroblasts and neurosphere derivation from embryonic mice brain provided high-resolution insights into the pathogenesis of SLOS, but are specific to the patient's mutations (80, 81). Furthermore, dhcr7^−/−^ zebrafish show neurological impairments, similar to those seen in SLOS patients, but cholesterol is readily available to them during development, comprising as much as 40% of the yolk (82, 83).

An accessible animal model is critical to further investigations of the pathophysiology of the disease as well as potential novel SLOS therapies. Today, cholesterol supplementation remains the standard treatment to ameliorate damage from SLOS, yet it does not rescue the DHCR7-dependent phenotypes. In contrast, the inhibition of oxysterol formation through antioxidant approaches has been proposed as a more promising avenue (67). Thus, antioxidant (mainly vitamin E) supplementation was also proposed (84), but such treatment is far from targeted therapy. Our findings reveal a naturally occurring, easily replicable framework with selective *DHCR7* downregulation and 7-DHC accumulation, extending in time for at least two-thirds of the equine pregnancy. The underlying mechanism of selective downregulation of *DHCR7* may still point researchers to unknown metabolic pathways that result from or accommodate this change, leading to a novel therapeutic approach to SLOS. The development of the DHCR7-deficient interstitial compartment of equine fetal gonads may offer an unusual opportunity to both generate new insights into the pathology of SLOS and serve as a model for drug development and testing. Future studies will require additional detailed untargeted and targeted metabolomic profiling, integrated with single-cell transcriptomics, to directly link sterol intermediates with steroid output within the cell populations of the equine fetal gonad interstitium. We further aim to investigate whether oxysterols are present in the equine fetal gonad, inducing apoptosis, and whether preventing their formation could alter the developmental trajectory of the gonad. This natural equine model avoids issues like embryonic lethality that have hampered laboratory studies to date. Furthermore, B-ring unsaturated steroid synthesis, and therefore response to potential treatments to ameliorate the effects of 7-DHC accumulation, can be monitored noninvasively, providing novel approaches to advance SLOS research efforts.

## Materials and methods

### Animals

Healthy, pregnant mares (*Equus caballus*, *n* = 21) were enrolled in the present study. All procedures were completed following the Institutional Animal Care and Use Committee of the University of Kentucky (Approval No. #2014-1341). Euthanasia was performed using sodium pentobarbital overdose, in accordance with the American Veterinary Medical Association guidelines. The gravid uteri were removed, and fetal gonads were collected in their entirety immediately after. The fetuses were grouped as follows: 4th GM (*n* = 4), 6th GM (*n* = 4), 9th GM (*n* = 5), 10th GM (*n* = 3), and 11th GM (*n* = 5). Additionally, fetal adrenal glands from 9th GM (*n* = 4), 10th GM (*n* = 2), and 11th GM (*n* = 4) were collected. Part of each sample was preserved in RNAlater™ (#AM7021; Invitrogen, Carlsbad, CA, USA), kept at 4 °C overnight, and then preserved at −80 °C until RNA isolation; part was snap frozen, and the other fixed in formaldehyde (10%) for 24 hours and stored in methanol (100%) until further processing. Sex of the fetuses (12 males, 9 females) was determined based on fetal morphology and confirmed by *INSL3* (Leydig cell biomarker (85)) and *AMH* (Immature Sertoli cell biomarker (86)) gene expression in the gonad.

### RNAseq

#### Total RNA extraction

Total RNA was extracted using the RNeasy® Mini Kit (Qiagen, Germantown, MD, USA). Sample quality was evaluated using the TapeStation system with the High Sensitivity D5000 Screen Tape Assay (Agilent Technologies, Santa Clara, CA, USA), and RNA quantification was carried out with the Qubit 2.0 RNA HS Assay (ThermoFisher Scientific, Waltham, MA, USA).

#### Library preparation and RNA sequencing

Poly(A)+ transcripts were isolated from total RNA using paramagnetic beads coupled with oligo(dT)25, following the NEBNext® Poly(A) mRNA Magnetic Isolation Module protocol (New England BioLabs Inc., Ipswich, MA, USA). Before the first-strand synthesis, samples were randomly primed with 5′-d(N_6_)-3′ primers [N = A, C, G, T] and fragmented according to the manufacturer's guidelines. First-strand cDNA synthesis was performed using ProtoScript® II Reverse Transcriptase with an extended incubation of 30 minutes at 42 °C. Next, library preparation continued with the NEBNext® Ultra™ II Non-Directional RNA Library Prep Kit for Illumina® (New England BioLabs Inc.), including end-repair, adaptor ligation, and amplification steps. Final library concentration was measured using Qubit 2.0 Fluorometer (Thermo Fisher Scientific), and quality was assessed using the Agilent TapeStation with the HSD1000 ScreenTape system (Agilent Technologies Inc.). Sequencing was performed on an Illumina® NovaSeq 6000 system (Illumina, San Diego, CA, USA) in a 150 bp paired-end, stranded configuration. The RNA-seq libraries contained an average of approximately 3.56 × 10^9^ nucleotides per sample, with a median insert size of ∼378 bp (±41.4 bp). Each sample had an average of 50 943 614 (±6 795 360) raw reads. After alignment and assignment to exonic regions, each sample had an average of 39 232 992 (±4 888 336) assigned fragments, representing the effective exon sequencing depth for downstream analyses. The RNA-seq data generated in this study have been deposited in the NCBI Sequence Read Archive database under project number PRJNA1305400.

#### Bioinformatics analysis

Raw sequencing reads were quality-trimmed, and adapter sequences were removed using Trim-Galore (v0.6.10, Babraham Bioinformatics, Cambridge, UK). Processed reads were aligned to the equine (*E. caballus*) reference genome assembly (TB-T2T, GCF_041296265.1) using STAR aligner v2.7.11a (87). Quality control steps before and after filtering and mapping used FASTQC v0.12.1 and MULTIQC v1.23 (88). Samools v1.22 (89) flagstat was used to output additional sequence alignment metrics.

Gene-level alignment quantification used featureCounts (90) v2.0.6. Differential expression analysis DESeq2 v1.46.0 in (Differential Expression analysis using the negative binomial distribution; R v4.4.2 and Rstudio v2025.06.99) was used, considering sex as a covariate. Comparisons were made as follows (Individual Pairwise Comparison vs Reference GM): 6th vs 4th GM; 9th vs 4th GM; 10th vs 4th GM; 11th vs 4th GM; 9th vs 6th GM; 10th vs 6th GM; 11th vs 6th GM; 10th GM vs 9th GM; 11th vs 9th GM; 11th vs 10th GM.

DEGs were identified using a false discovery rate (FDR) adjusted *P*-value < .01 and a minimum log_2_ fold change ≥ |2| threshold. The DGE.Tools2^10^ convertCounts tool was used to convert raw counts to transcript-per-million (TPM) relative abundance values. Principal component analysis (PCA) was performed using R stats v3.6.2 prcomp library and visualized using ggplot2v3.5.2 (91) in R v4.4.2, based on TPM values. Heatmaps of gene-wise *z*-score normalized TPM values were generated using pheatmap v1.0.13. Descriptive statistical analysis was performed in JMP® Pro 18 (SAS Institute Inc., Cary, NC, USA). Gene ontology analysis was performed using ShinyGO 0.82 (92).

#### Multiplex immunofluorescence for CYP11A1 and CYP17A1

Four-micron sections of formalin-fixed, paraffin-embedded (FFPE) tissues were mounted on positively charged Superfrost® Plus Slides (VWR, Radnor, PA, USA) and subjected to tyramide signal amplification–mediated multiplex immunostaining using the automated BOND RXm platform (Leica Biosystems, Deerpark, IL, USA) and Akoya Bioscience's Opal™ dyes (Akoya Biosciences, Marlborough, MA, USA). Briefly, tissue sections were subjected to automated baking and deparaffinization followed by an initial automated heat-induced antigen retrieval (HIER) step using a ready-to-use EDTA-based solution (pH 9.0; Leica Biosystems) for 20 minutes at 100 °C. Following the retrieval step, tissue sections were blocked for 5 minutes at room temperature with a ready-to-use antibody block solution (Akoya Biosciences) and subsequently incubated with the first primary antibody (rabbit anti-CYP11A [Sigma Aldrich, HPA016436, RRID:AB_1847423] diluted 1:500 in antibody diluent/block) for 30 minutes at room temperature. Following automated washing steps, tissue sections were incubated with an HRP-conjugated, polymer-labeled goat anti-rabbit IgG (PowerVision, Leica Biosystems) diluted 1:1 in 1X phosphate-buffered saline for 10 minutes at room temperature. Signal was subsequently developed by incubating with Opal™ 520 (1:150 in 1X Amplification diluent, Akoya Biosciences) for 10 minutes at ambient temperature. A sequential round of staining was performed as indicated above and after a stripping step using a ready-to-use citrate-based pH 6.0 solution (Leica Biosystems) for 20 minutes at 100 °C. A rabbit anti-CYP17A1 antibody (93) (diluted 1:4000 in antibody diluent/block; RRID:AB_2491005) was applied for 30 minutes at room temperature, followed by the aforementioned goat anti-rabbit and incubation with Opal™ 570 (1:150 in 1X amplification diluent, Akoya Biosciences) for 10 minutes at room temperature. Tissue sections were finally counterstained using DAPI (Akoya Biosciences) for 5 minutes at room temperature and coverslipped with Prolong® Diamond AntiFade Mountant (ThermoFisher Scientific). Tissue sections were subsequently imaged using Akoya Bioscience's PhenoImager HT 2.0 with onboard spectral unmixing. Regions of interest were captured with PhenoChart 2.0.0 and Inform 3.0. Adult equine adrenal cortex was used as a positive control, and adrenal medulla was used as a negative control for both antibodies.

#### RNAscope™ in situ hybridization (ISH) for DHCR7 mRNA

For RNAscope™ ISH, a probe targeting nt 317-1389 of *E. caballus* DHCR7 mRNA (XM_023654657.1; Cat. No. 1596748-C1) was designed and synthesized by Advanced Cell Diagnostics (ACD, Newark, CA, USA). The RNAscope™ ISH assay was performed using the RNAscope 2.5 LSx RED Reagent Kit (ACD) on the automated BOND RXm platform (Leica Biosystems) as described previously (94). Briefly, four-micron sections of FFPE tissues were mounted on positively charged Superfrost® Plus Slides (VWR) and subjected to automated baking and deparaffinization followed by HIER using a ready-to-use EDTA-based solution (pH 9.0; Leica Biosystems) at 100 °C for 15 minutes. Subsequently, tissue sections were treated with a ready-to-use protease (RNAscope® 2.5 LSx Protease, ACD) for 15 minutes at 40 °C, followed by a ready-to-use hydrogen peroxide solution for 10 minutes at room temperature. Slides were then incubated with the ready-to-use probe mixture for 2 hours at 40 °C, and the signal was amplified using a specific set of amplifiers (AMP1 through AMP6 as recommended by the manufacturer). The signal was detected using a Fast Red solution for 10 minutes at room temperature. Finally, slides were counterstained with a ready-to-use hematoxylin for 5 minutes, followed by 5 washes with 1X BOND Wash Solution (Leica Biosystems). Slides were rinsed in deionized water, dried in a 60 °C oven for 30 minutes, and mounted with Ecomount® (Biocare, Concord, CA, USA). Slides were scanned on the PhenoImager HT 2.0 and analyzed using QuPath 0.5.1.

#### Histopathology and immunohistochemistry

H&E staining was performed according to standard protocols on samples prepared as above. For IHC, 4-μm sections of FFPE tissues were mounted on positively charged Superfrost® Plus slides and subjected to IHC using the automated BOND RXm and the Polymer Refine Detection kit (Leica Biosystems). Following automated deparaffinization, HIER was performed using a ready-to-use citrate-based buffer (pH 6.0) or an EDTA-based buffer (pH 9.0) at 100 °C for 20 minutes before incubation with primary antibodies (Table 1). Sections were then incubated with the primary antibodies (dilutions listed in Table 1) for 30 minutes at room temperature, followed by either a polymer-labeled goat anti-rabbit or anti-mouse IgG coupled with horseradish peroxidase for 8 minutes at room temperature. 3,3′-diaminobenzidine tetrahydrochloride was used as the chromogen (10 minutes), and counterstaining was performed with hematoxylin. Slides were mounted with a permanent mounting medium.

**Table 1:** List of antibodies and concentrations used in this study

	Clone	Species	Dilution	Retrieval	Manufacturer
**Pan-cytokeratin**	AE1/AE3	Mouse	1:200	EDTA-based, 20 minutes	Dako RRID:AB_2132885
**Vimentin**	V9	Mouse	1:500	Citrate-based, 20 minutes	Leica Biosystems RRID:AB_564055
**Smooth muscle actin**	1A4	Mouse	1:400	EDTA-based, 20 minutes	Dako RRID:AB_2223500
**Desmin**	D33	Mouse	1:50	None	Dako RRID:AB_2335684

#### Gas chromatography–mass spectrometry

Cholesterol and 7-dehydrocholesterol (7-DHC) were extracted from tissue samples (*n* = 29) following a previously published method with minor modifications (95). Approximately 50 mg of tissue was used for each sample. Antioxidant solution containing 50 µg/mL butylated hydroxytoluene and 200 µg/mL triphenylphosphine in ethanol was added at a ratio of 10 µL/mg tissue. Samples were homogenized with stainless steel beads using a GenoGrinder 2010 (Spex Sample Prep, Mutchen NJ) at 1500 rpm for 6 minutes. Homogenized samples were chilled for 30 minutes at −20 °C to facilitate protein precipitation and then centrifuged at 10 000 × g for 5 minutes at 4 °C. For most samples, a 50 µL subaliquot of the 10-fold diluted supernatant was transferred to a new tube and enriched with cholesterol-d7 to achieve a final concentration of 1 µg/mL. For 10 adrenal samples, a 100-fold dilution was required to bring cholesterol into our calibration range. Internal standard spiked samples were extracted with 400 µL n-hexane by vortex mixing, followed by centrifugation at 10 000 × *g* for 2 minutes at 4 °C. The upper organic layer was transferred and evaporated to dryness. Dried extracts were derivatized with 100 µL *N,O*-Bis(trimethylsilyl)trifluoroacetamide at 60 °C for 30 minutes. Derivatized samples were transferred to flat-bottom glass inserts in 2 mL GC vials, capped, and analyzed by GC–MS immediately.

GC–MS analysis was performed on a Pegasus BTX benchtop GC-TOFMS system (LECO Corp., St. Joseph, MI). Each sample (0.5 µL) was injected in splitless mode at 275 °C and separated on a 30 m × 0.25 mm i.d., 0.25 µm Rtx-5Sil MS capillary column (Restek, Bellefonte, PA). Helium was used as the carrier gas at a constant flow rate of 1 mL/min. The GC oven temperature was initially held at 50 °C for 1 minute, ramped at 20 °C/min to 330 °C, and held for 5 minutes. Electron ionization was performed at 70 eV with the ion source temperature of 280 °C. Mass spectral data were acquired in full-scan mode over *m/z* range of 85 to 500. Raw GC-MS data files (.cdf) were converted to.D file format and processed using MassHunter software. Quantification was based on the peak area ratios obtained from extracted ion chromatograms of 7-DHC, CHOL, and CHOL-d7 at *m/z* 351, 329, and 336, respectively. Analytes were quantified using a 7-point calibration curves ranging from 0.05 to 25 μg/mL for cholesterol and 0.01 to 25 μg/mL for 7-DHC with quadratic regressions and 1/x weighting and bracketed all reported samples. Calibration accuracies were within 87% to 112%. Instrument performance was monitored by analyzing a mid-level calibration standard as a quality control sample after every eight injections. Quality control reproducibility was acceptable, with %CV values of 2.39% for cholesterol and 2.84% for 7-DHC. Differences in 7-DHC abundance across GMs were analyzed by one-way ANOVA using JMP® Pro 18 (SAS Institute Inc.), followed by Tukey's post-hoc multiple-comparison testing. The relationship between DHCR7 expression (TPM) and 7-DHC concentration (mg/g tissue) was evaluated using Pearson correlation analysis in JMP® Pro 18 (SAS Institute Inc.). Correlation strength was reported as Pearson's correlation coefficient (*r*).

Representative chromatograms, calibration curves, and mass spectra used for sterol identification are provided in Fig. S2 (96).

### Accession number(s)

The RNAseq data from this study were deposited in Sequence Read Archive under accession number PRJNA1305400.

## Data Availability

Original data generated and analyzed during this study are included in this published article or in the data repositories listed in References.

## Abbreviations

7-DHC: 7-dehydrocholesterol
AMP1–AMP6: RNAscope amplification steps 1–6
ANOVA: analysis of variance
AVMA: American Veterinary Medical Association
BHT: butylated hydroxytoluene
BSTFA: N,O-bis(trimethylsilyl)trifluoroacetamide
cDNA: complementary DNA
CHOL: cholesterol
CHOL-d7: deuterated cholesterol
CV: coefficient of variation
DAB: 3,3′-diaminobenzidine tetrahydrochloride
DAPI: 4′,6-diamidino-2-phenylindole
DEG: differentially expressed gene
DESeq2: Differential Expression Sequencing 2
DHCEO: 3β,5α-dihydroxycholest-7-en-6-one
DHCR7: 7-dehydrocholesterol reductase
DHEA: dehydroepiandrosterone
EDTA: ethylenediaminetetraacetic acid
FASTQC: Fast Quality Control
FFPE: formalin-fixed, paraffin-embedded
FDR: false discovery rate
GC-MS: gas chromatography–mass spectrometry
GM: gestational month
GO: Gene Ontology
HE: hematoxylin and eosin
HIER: heat-induced epitope retrieval
HRP: horseradish peroxidase
IF: immunofluorescence
IgG: immunoglobulin G
IHC: immunohistochemistry
INSL3: insulin-like peptide 3
ISH: in situ hybridization
KO: knockout
m/z: mass-to-charge ratio
miR: microRNA
NCBI: National Center for Biotechnology Information
PCA: principal component analysis
QC: quality control
RNA: ribonucleic acid
RNAseq: RNA sequencing
RRID: Research Resource Identifier
SLOS: Smith–Lemli–Opitz syndrome
SRA: Sequence Read Archive
STAR: Spliced Transcripts Alignment to a Reference
TPM: transcripts per million
TPP: triphenylphosphine
TSA: tyramide signal amplification.
